# Immediate Evaluation of the Effect of Infrared LED Photobiomodulation on Childhood Sleep Bruxism: A Randomized Clinical Trial

**DOI:** 10.3390/life12070964

**Published:** 2022-06-27

**Authors:** Fernanda Yukie Kobayashi, Paula Midori Castelo, Fabiano Politti, Monise Mendes Rocha, Rafael Zaratin Beltramin, Mônica Da Consolação Canuto Salgueiro, Marcela Leticia Leal Gonçalves, Samir Nammour, Aldo Brugnera Júnior, Ravana Angelini Sfalcin, Sandra Kalil Bussadori

**Affiliations:** 1 Postgraduation Program in Biophotonics Applied to Health Sciences, Universidade Nove de Julho (UNINOVE), São Paulo 01525-000, Brazil; fernandaykobayashi@gmail.com (F.Y.K.); ravanasfalcin@hotmail.com (R.A.S.); 2 Department of Pharmaceutical Sciences, Universidade Federal de São Paulo (UNIFESP), Diadema 04021-001, Brazil; paula.castelo@unifesp.br; 3 Postgraduation Program in Rehabilitation Sciences, Universidade Nove de Julho (UNINOVE), São Paulo 01525-000, Brazil; politti@uni9.pro.br (F.P.); monisedentista@gmail.com (M.M.R.); beltraminzaratin@gmail.com (R.Z.B.); monicasalgueiro@globo.com (M.D.C.C.S.); 4 Postgraduation Program in Health and Environment, Universidade Metropolitana de Santos (UNIMES), Santos 11045-002, Brazil; marcelalleal@hotmail.com; 5 Director of the Post-Graduate: Laser Application in Dental Medicine, Department of Dental Sciences, Faculty of Medicine, University of Liege, 4000 Liège, Belgium; s.namour@uliege.be; 6 National Institute of Science and Technology, INCT “Basic Optics Applied to Life Sciences”, IFSC, USP, São Carlos 13566-590, Brazil; aldobrugnera@gmail.com

**Keywords:** bruxism, saliva, laser therapy, muscles, electromyography, dopamine

## Abstract

The gold standard for the management of sleep bruxism (SB) is the use of a rigid occlusal splint; however, there are limitations for its use in children and alternatives to the management of SB are needed. Photobiomodulation therapy has been used with positive results in temporomandibular disorders. This study aimed to evaluate the effects of photobiomodulation therapy with infrared LED in children with SB. Thirty children were divided into three groups: Group 1: control/absence of bruxism (*n* = 10); Group 2: SB treated with infrared LED (*n* = 10); Group 3: SB treated with occlusal splint (*n* = 10). Electromyographic evaluation of masseter, anterior temporalis, and upper trapezius, and salivary dopamine levels were assessed before and after treatments. Data were statistically analyzed using two-way mixed model ANOVA. An increase in the temporalis and right masseter EMG activity at rest was observed in Group 3, with large effect size (*p* < 0.05). Left masseter and temporalis EMG activity did not differ over time in the LED group, similar to the control group. Moreover, the EMG activity of masticatory muscles during chewing and upper trapezius muscle did not differ over time in all groups. The results also pointed to a difference in the levels of dopamine between children with and without SB, with Group 3 showing higher levels in the pre-treatment time compared to controls (*p* < 0.025). In conclusion, an increase in the masticatory muscles activity at rest was observed in children undergoing splint therapy. Moreover, a difference in the levels of salivary dopamine was found between children with and without SB.

## 1. Introduction

According to the last *International Consensus on the Assessment of Sleep Bruxism*, sleep bruxism (SB) is defined as a masticatory muscle activity that occur during sleep, characterized as rhythmic (phasic) or non-rhythmic (tonic) and should not be considered as a movement or a sleep disorder in healthy individuals [1]. Clenching and/or grinding the teeth while sleeping is named as primary bruxism or SB, while clenching/grinding the teeth during the day is named as secondary or awake bruxism [1].

Under the control of the central nervous system, the increase in masticatory muscle activity is the primary SB signal. It can be detected by polysomnography, an exam considered as the gold standard for SB diagnosis in adults [1]. In children, electromyography (EMG) is a parameter that has been used in patients with bruxism, both for the assessment the normal aspects of masticatory muscles or as a follow-up of interventions [2,3,4], once the use of polysomnography for the diagnosis of SB in children has many limitations, such as its high-cost and child’s lack of collaboration. The etiology of SB in children remains unknown, and the prevalence found in the literature is variable due to the different diagnostic techniques applied; according to a recent systematic review, “possible” and “probable” SB affects one in four Brazilian children [5]. Previous studies investigated the impact that emotional disturbances may have on triggering SB, especially those related to the worse symptoms (pain, tooth wear). A previous study [6] reported that bruxism may be related to psychological factors, while stressed individuals are described as being more susceptible to develop SB [7].

An important neurotransmitter mediated by the central nervous system is the dopamine, that belongs to the family of catecholamines and phenylethylamines, whose functions are related to movement control, learning, mood modulation, emotions, cognition, and information storage [8]. Dopamine can be quantified based on salivary levels [7] and changes in dopamine levels have been linked to neuromuscular disorders, such as Parkinson’s disease, in which decreased concentrations of dopamine are found [9]. As the contribution of single nucleotide polymorphisms (SNPs) in dopaminergic pathway to bruxism development has been previously proposed [10] and no previous study investigating the salivary concentrations of dopamine in children with bruxism was found, the quantification of dopamine changes over time during SB treatment is of interest.

The environment may have an influence on the reflexes of the body in response to a previous stressor. The effects related to the surrounding environment are processed and evaluated by the central nervous system, especially by the limbic system and hypothalamus, which lead to an adequate emotion reaction and stimulate the sympathetic nervous system, releasing adrenaline that triggers the increase in the respiratory and heart rates, muscle tension, glucose level, and blood pressure. In this way, any information that provokes such a response is recognized as a stressor [11,12]. Furthermore, the effects of the suppression of emotions and motor activities may overload the function of the organism, resulting in several neuromuscular disorders [13]. In some situations, SB has been associated with anxious behaviors [14,15] and it is very common to observe anxiety in children, especially in pediatric clinical practice.

According to da Silva et al. (2015) [16], the recommended treatment for bruxism is the use of a rigid occlusal splint (Michigan splint). Some alternative treatments for muscle disorders, such as the photobiomodulation in masticatory muscles have been developed and showed satisfactory results. Photobiomodulation has been used in acupuncture points for adults with temporomandibular disorder [16], with a significant difference found in TMD signs and symptoms after application of the technique. Physiologically, photobiomodulation stimulates cells and increases blood circulation, vasodilatation, analgesia, and has anti-inflammatory effects, such as the decrease in edema and acceleration of the healing process of injured tissues. Moreover, photobiomodulation therapy with light-emitting diode (LED) has also been used for the treatment of muscle disorders. A previous treatment protocol for fibromyalgia including the use of LED has been proposed [16], which considered LED application as an accessible treatment due to its low cost and more durable device, making the proposed treatment an alternative and viable option.

Thus, the aim of this study was to evaluate the effects of photobiomodulation on masticatory muscles activity and salivary dopamine levels in children with SB, before and immediately after therapy and comparing them to the rigid occlusal splint use and a control group.

## 2. Materials and Methods

This randomized clinical trial was approved by the Research Ethics Committee of the Nove de Julho University (approval protocol number: 1.333.636) and registered at the Clinical Trials Database ID NCT 03710174. The sample size was calculated considering α = 0.05 and an 80% of patient’s adherence, in which 20% could be considered as possible patients with non-adherence, totaling 30 individuals (*n* = 10 in each group). The sample size calculation was based on the results of a previous study which evaluated the decrease in signs and symptoms of TMD after occlusal splint therapy [17].

### 2.1. Inclusion Criteria

Children with mixed dentition stage (permanent incisors and first molars erupted) was included in the study.

### 2.2. Exclusion Criteria

Children with dental caries or using medications such as anti-inflammatory drugs, corticoids, muscle relaxants, antidepressants, or anticonvulsants were excluded from the study. In addition, children presenting chronic diseases which might affect muscles or motor coordination, and no collaborator children were also excluded.

### 2.3. Allocation Procedure

**Figure 1:**
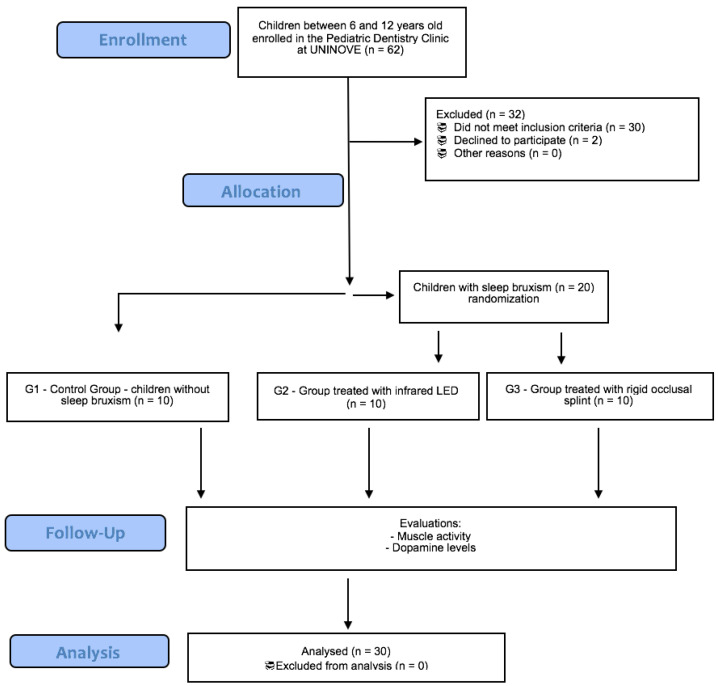
Flowchart with the description of participants allocation.

The randomization was performed for the groups with SB using sequentially numbered opaque envelopes, as described in the flowchart (Figure 1). A computer program was used to randomly determine the contents of the envelopes. Each envelope had a sheet of paper indicating the group to which the participant would be allocated. The envelopes were properly closed until the time of treatment.

### 2.4. Groups

The participants were allocated in the following groups:

- Group 1 (*n* = 10): control—absence of SB (4 girls; mean age 7.0 ± 0.9 years);
- Group 2 (*n* = 10): children with SB treated with infrared LED (5 girls; mean age 7.1 ± 1.1 years);
- Group 3 (*n* = 10): children with SB treated with occlusal splint (7 girls; mean age 7.6 ± 1.3 years).

### 2.5. Clinical Evaluation

A trained and calibrated dentist performed the bruxism diagnoses. Two other dentists were responsible for the application of photobiomodulation, occlusal splint treatment, and salivary analysis. A fourth dentist performed the electromyography analysis and statistics. The period of assessment to complete all steps was of 4 months.

The diagnosis of SB was based on the report of the child’s guardians/parents, regarding the occurrence of teeth grinding. The child’s guardians/parents fulfilled a questionnaire reporting the child’s medical and dental history along the study, and then, delivered back to the school for subsequent evaluation. In addition, the following clinical signs were also observed: abnormal tooth wear on the functional cusps of the teeth, dental marks on the tongue, alba line on the oral mucosa along the occlusal plane, gingival recession, mandibular and/or maxillary torus, and fractures and/or cracks in the teeth [14]. In this way, only children with “probable” SB, that is, report of the child’s guardians/parents and clinical signs, were included, as stated by Lobbezoo et al. (2018) [1].

Group 1 (control) included healthy children without SB that did not receive any intervention; they were evaluated two times with an interval of 30 days (similarly to Group 3/Splint) for the collection of data on electromyographic analysis and salivary dopamine levels.

### 2.6. Photobiomodulation Treatment

**Figure 2:**
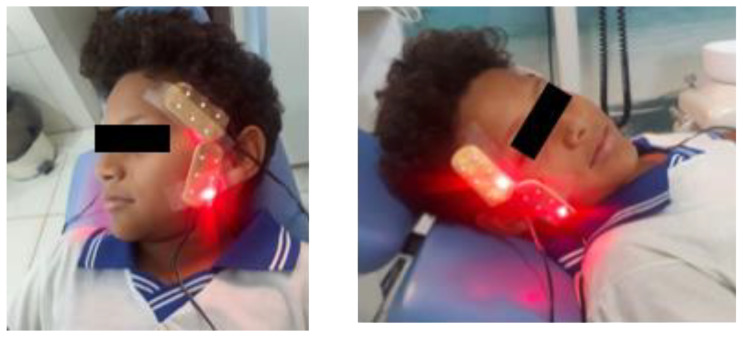
Light-emitting diode (LED) positioning over the masseter and anterior temporalis muscles.

Group 2 underwent the photobiomodulation treatment with initial and immediate post-treatment evaluations of salivary dopamine and electromyographic activity. For this purpose, an infrared LED device was used (Odontolux model, Cosmedical^®^, Davie, FL, USA, https://www.cosmedical.com.br/odontollux accessed on 1 March 2018) (3 × 6 cm), using a board with 6 LEDs with a wavelength of: 850 nm ± 20 nm, optical spot of 5 ± 2 mm, with a dose of 2.675 J/cm^2^, and optical output of 2~5 mW [18]. The LED device was positioned in the temporal and masseter muscles. Irradiation was performed during 7 min in each muscle, as shown in Figure 2. A single photobiomodulation session was conducted.

### 2.7. Occlusal Splints Treatment

Group 3 was treated with a standard protocol of a rigid occlusal splint. After the initial evaluation of muscle activity and saliva collections, maxillary and mandibular stone casts were obtained from patients for further fabrication of the rigid occlusal appliance delivered to the participants one week later. Written and verbal instructions for use were given. On the 30th day, after wearing the splints for at least 12 h/day, the participants attended the dental appointment when another electromyographic assessment and saliva collection were performed.

### 2.8. Electromyographic Assessment

The electromyographic evaluation (EMG) was carried out in the masseter, anterior temporalis, and upper trapezius muscles (BTS TMJOINT). The participant was positioned according to the Camper’s plane, parallel to the floor. Measurements were taken three times on both sides (left/right) with the muscles at rest, habitual maximum intercuspation (isometric contraction), and chewing on a Parafilm^®^ (isotonic contraction) [2,19]. The signal was captured for 10 s under each condition. The first chewing cycle was excluded, and the subsequent five cycles were collected. Further, the signal was normalized considering the maximum voluntary contraction (%MVC).

### 2.9. Salivary Dopamine Levels

The participants and their guardians/parents received verbal and written instructions to avoid any physical activity, ingestion of substances with alcohol or caffeine, soft drinks, tea, and chewing gum in the 24 h before the collection of the saliva. Samples were collected in the morning, using polyester swabs (Salivette^®^, Sarstedt, Germany). The polyester swab was placed under the tongue and the child was instructed to move it to collect the stimulated saliva, which was cooled immediately after collection [18]. Samples with visible signs of blood were excluded due to the possible contamination by plasma, and consequently change the parameters [20]. The samples were then centrifuged at 3500 rpm for 5 min and stored at −40 °C. Dopamine levels were determined using an enzyme-linked immunosorbent assay (Dopamine Research ELISA BA E-5300 by Salimetrics, State College, PA, USA). The samples were thawed and centrifuged again. The sample volume was at 25 μL and the incubation time was at 60 min. The enzyme-linked immunosorbent assay kit plates also had controls and standards. Thus, there was a competition between an unlabeled antigen and an enzyme-labeled antigen for a specific number of binding sites on the antibody. The analysis involved reading of the solution absorbance using a microplate reader set at 450 nm and a 630 nm correction filter, following the manufacturer’s instructions [18,21]. The salivary test was chosen because it is a non-invasive, pain-free method, suitable to be performed in a school environment and well accepted at this age.

### 2.10. Statistical Analysis

The statistical analysis was performed using SPSS version 28.0 (SPSS Inc., Chicago, IL, USA). Means and standard deviation were used for descriptive purposes. The one-way ANOVA was used to test the variables age, EMG data and salivary dopamine levels at baseline (pre-treatment). A general linear model—two-way mixed model ANOVA—was used to test the effects of within-subjects factor (time: pre- and post-treatment) and the between-subjects factor (group: control, LED, and splint) and the interaction time × group effect on the observed variance of the EMG data and dopamine levels (considered as dependent variables). The effect size (partial *eta* squared) was also obtained. The results of the Box’s test, Mauchley’s sphericity test, and Levene’s equality of variances were evaluated as assumptions; when necessary, the Huynh–Feldt correction was applied. The interpretation was based on the values established by Cohen (1988) [22]: small effect (range η^2^ = 0.01–0.06), moderate effect (η^2^ = 0.06–0.14), and large effect (greater than η^2^ = 0.14). For all analysis, a value of *p* < 0.05 was considered as significant.

## 3. Results

The three groups were homogeneous for the variable ‘age’ (*p* = 0.464). Table 1 shows the means and standard deviation of the normalized EMG activity of masticatory muscles at rest position and chewing and the upper trapezius muscle, and the effects of time (pre- and post-treatment) and time X group interaction.

According to the results, a time effect was observed for the right masseter EMG activity at rest, and all groups showed a significant increase in right masseter activity after treatment with large effect size. Moreover, a significant interaction effect time X group was found for the left masseter, left temporalis, and right temporalis activity at rest, in which a significant increase in the EMG activity was found only in the Group 3 (rigid occlusal splint use), with a large effect size.

Considering the EMG activity of masticatory muscles during chewing, no significant difference was observed over time in all groups. Moreover, no significant difference was observed in the upper trapezius EMG activity.

At baseline (pre-treatment), the salivary level of dopamine was different between groups, with Group 3 (occlusal splint—with SB) showing higher levels than the control group (Group 1—without SB) (F = 4.320; *p* = 0.025).

**Figure 3:**
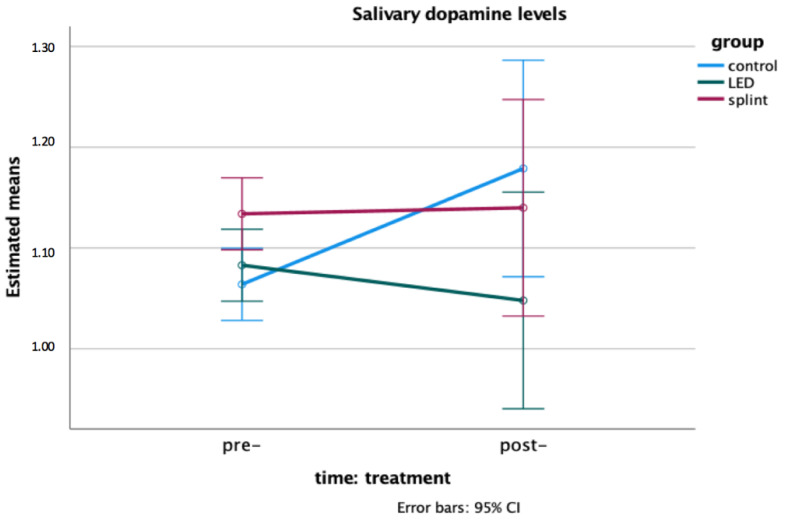
Interaction effect time X group on the salivary levels of dopamine: a two-way mixed model ANOVA. No significant time (*p* = 0.377) or time X group effect (*p* = 0.159) was found. A difference was found between Groups “control” and “LED” in the pre-treatment time (*p* = 0.025). Means and confidence intervals are shown (ng/mL).

However, no significant time or time X group effect was observed for the salivary dopamine levels as shown in Figure 3, that is, no significant change was observed before and after treatment (*p* > 0.05).

## 4. Discussion

In this clinical trial, three groups of children were compared: two groups of children with SB that were submitted to photobiomodulation and rigid occlusal splints, respectively, and a control group of children without SB that did not receive any intervention. The results showed that, while the right masseter muscle activity at rest increased after intervention in all groups, the temporalis (right and left) and left masseter EMG activity increased only in the group of children who underwent the use of occlusal splint. In addition, salivary dopamine levels did not differ over time in the three groups, but a difference was observed in the pre-treatment levels between the group of children with SB that used occlusal splints and the control group (without SB).

Alternative therapies for bruxism management have been proposed, beyond the use of occlusal splints considered as the gold standard, due to the high frequency of patients’ complaints. These alternatives include drugs such as buspirone [23], fluoxetine [24], and diazepam [25], although these studies observed an inefficacy with the pharmacological therapy and emphasized their potential adverse/side effects. In an effort to search other non-pharmacological methods, our study evaluated the effect of photobiomodulation, a non-pharmacological therapy, on masticatory muscle activity and salivary dopamine levels to contribute to the evaluation of the efficacy and biosafety of LED photobiomodulation on the management of bruxism.

The application of LED to the masticatory muscles affects the local tissues around the light application site and previous studies have shown the effectiveness of LED treatment in muscle tissues [26,27,28]. The literature also reports that it is possible to observe systemic responses through light therapy, with the action of deep light stimulation directly on the brain [29]. Another application of the method involves the intravascular laser irradiation for blood therapy, which has already shown good results [30,31].

The effects of photobiomodulation therapy have already been evaluated in muscle disorders, such as temporomandibular disorders, and obtained good results. In a single combined phototherapy session, a reduction in the intensity of pain in individuals with temporomandibular disorders was observed. Photobiomodulation therapy combined with two light sources (LED and laser) has shown to be useful intervention for people with TMD. This mode of photobiomodulation is another option that may assist the rapid intervention of pain symptoms, promoting a considerable degree of patient comfort moments after its application [32].

While a decrease in the EMG of masseter and temporalis at rest and clenching positions was observed after splint therapy in the study of Akat et al. [33], in the present study an increase in the temporalis and masseter EMG activity at rest was observed in group of children who underwent occlusal splint therapy. It is important to point out that the former study compared raw EMG data, while the present study compared normalized EMG data (%MVC). Still, another study showed that EMG activity of the masseter muscle recorded for four consecutive nights did not change with the use of Michigan splint [34]. Considering that hyperactivity, fatigue, muscle spasms, myofascial pain, and morphological and functional changes in the masticatory system are symptoms reported in patients with bruxism [35] and the use of occlusal splint causes an increase in the vertical dimension that could have a muscle relaxant effect, the observed increase in masseter and temporalis EMG activity at rest was an unfavorable and not expected result in Group 3 (splint). On the other hand, the right/left temporalis EMG activity at rest did not differ over time in the LED group, similar to the control group. Moreover, the EMG activity of masticatory muscles during chewing and upper trapezius muscle did not differ over time in all groups.

The results also suggested a difference in the salivary levels of dopamine between children with and without SB, with the children enrolled in the occlusal splint group showing higher levels in the pre-treatment time compared to those without SB. It is known that many neurochemicals (e.g., serotonin, dopamine, gamma aminobutyric acid, noradrenaline) may be involved in both the genesis of rhythmic jaw movements and the modulation of muscle tone during sleep [36]. A case series of Chen et al. (2005) [37] with three patients with diurnal/nocturnal bruxism showed that they differed from the usual features of nocturnal bruxism in hypoperfusion of the left frontal lobe, poor response to L-dopa or bromocriptine therapy and a favorable response to metoclopramide; the authors also hypothesized that hypersensitive presynaptic dopamine receptors might be the underlying pathology responsible for this type of bruxism. However, due to the small number of individuals included in our study (*n* = 10 each group), this finding should be considered with caution and future studies should be encouraged to ascertain the role of dopamine in the SB etiology and the mechanism by which drugs that act on its receptors can trigger or attenuate episodes of bruxism [38].

Although the occlusal splint has been considered the gold standard for the management of bruxism [34], the adherence to the treatment by the children and their parents is challenging in the clinical practice; therefore, the development of new strategies is necessary since SB may be associated with a negative impact on sleep quality and functional alterations [35].

Despite the new and important results that the study brings, there are some limitations that should be mentioned. The evaluation of the child’s perception of each therapy, as well the oral health quality of life, would improve the understanding of the changes observed during the trial. However, due to the many other clinical and research steps foreseen in the study, including another one would be time consuming and exhausting for children.

## 5. Conclusions

While the right masseter muscle activity at rest increased after intervention in all groups, the temporalis (right and left) and left masseter EMG activity at rest increased only in the group of children who underwent the use of occlusal splint. In addition, salivary dopamine levels did not differ over time in the three groups, but a difference was observed in the pre-treatment levels between the group of children with SB which used occlusal splints and the control group (without SB). This study contributes to the evaluation of photobiomodulation as a low-cost, easy-to-apply option in the management of muscle disorders related to bruxism.

## Figures and Tables

**Table 1:** Mean (±SD) of the amplitude of the electromyographic signal of the masticatory muscles (right temporal, right masseter, left temporal, and left masseter) and upper trapezius muscle normalized by the maximum voluntary contraction (%MVC in µV) and the results of time and group effects: a two-way mixed model ANOVA.

	Control Group		LED Group		Occlusal Splint Group		Two-Way Mixed Model ANOVA		
	Pre	Post	Pre	Post	Pre	Post	*p*-Value	*eta* Partial Squared	F
**Rest position**									
**Right temporalis (%MVC)**	9.6 (6.3)	7.4 (5.6)	4.9 (4.3)	7.7 (6.4)	6.5 ^A^ (6.3)	13.8 ^B^ (10.5)	interaction effect: *p* = 0.019	0.314	4.807
**Right masseter (%MVC)**	8.1 ^A^ (4.8)	8.6 ^B^ (5.0)	5.1 ^A^ (3.6)	8.2 ^B^ (4.9)	8.6 ^A^ (6.2)	12.8 ^B^ (8.6)	time effect: *p* = 0.044	0.180	4.598
**Left temporalis (%MVC)**	9.4 (5.2)	7.3 (4.8)	5.3 (3.8)	8.8 (4.8)	6.0 ^A^ (4.8)	12.5 ^B^ (6.6)	interaction effect: *p* = 0.021	0.308	4.669
**Left masseter (%MVC)**	7.2 (3.0)	5.8 (3.4)	5.5 (2.8)	6.5 (3.9)	6.0 ^A^ (2.9)	9.6 ^B^ (5.2)	interaction effect: *p* = 0.037	0.270	3.888
**Chewing**									
**Right temporalis (%MVC)**	32.0 (13.5)	27.7 (14.0)	28.9 (12.5)	34.8 (25.9)	28.7 (17.8)	28.7 (15.9)	NS	-	
**Right masseter (%MVC)**	25.3 (18.9)	23.9 (13.0)	22.4 (12.8)	33.4 (34.4)	27.3 (12.5)	30.1 (12.6)	NS	-	
**Left temporalis (%MVC)**	34.3 (11.5)	28.9 (9.4)	35.5 (12.1)	37.6 (16.5)	27.9 (15.6)	31.4 (13.5)	NS	-	
**Left masseter (%MVC)**	23.9 (10.2)	23.3 (5.4)	22.6 (10.5)	26.9 (14.7)	23.9 (13.0)	25.8 (10.1)	NS	-	
**Arm elevation**									
**Upper trapezius (%MVC)**	36.2 (10.4)	36.5 (8.8)	35.0 (10.5)	28.1 (12.6)	38.4 (15.6)	32.6 (11.6)	NS	-	

A ≠ B (*p* < 0.05; Two-way Mixed Model ANOVA and post hoc test with Bonferroni correction). NS, not significant.

## Data Availability

The data presented in this study are available on request from the corresponding author.
